# Developing and Validating the English Teachers’ Cognitions About Grammar Teaching Questionnaire (TCAGTQ) to Uncover Teacher Thinking

**DOI:** 10.3389/fpsyg.2022.880408

**Published:** 2022-06-20

**Authors:** Lawrence Jun Zhang, Qiang Sun

**Affiliations:** ^1^Faculty of Education and Social Work, University of Auckland, Auckland, New Zealand; ^2^School of Foreign Languages, Henan Polytechnic University, Jiaozuo, China

**Keywords:** language education, teachers’ cognitions, grammar teaching, questionnaire development and validation, English as a foreign language (EFL), China

## Abstract

It is well-acknowledged that teachers play a significant role in enhancing student learning and that investigating teachers’ cognitions about teaching is a first and important step to understanding the phenomenon. Although much research into teachers’ cognitions about grammar teaching has been conducted in various socio-cultural contexts, little has been reported on cognitions of Chinese teachers of English as a foreign language (EFL) so far. Such understanding is of primary importance to student success in language learning given the sociocultural context where grammar takes a lion’s share in high-stakes examinations. In order to address this research gap, we developed and validated the Chinese EFL Teachers’ Cognitions about Grammar Teaching Questionnaire (TCAGTQ). Two subsamples (*n*1 = 314, *n*2 = 215) were randomly invited to respond to the TCAGTQ and the data were then subjected to exploratory factor analysis (EFA) and confirmatory factor analysis (CFA) to test the validity and reliability of the instrument. The EFA excluded eight items from the TCAGTQ and generated six factors with 27 items. The CFA result from the other subsample supported a six-factor model with a good model fit. Moderate correlations between the six factors also supported the predictive validity of the questionnaire, showing that the TCAGTQ is a valid and reliable inventory for measuring Chinese university EFL teachers’ cognitions about grammar teaching. Our findings suggest that the TCAGTQ can be used as a useful tool for teachers to self-assess their professional practice for improving teaching.

## Introduction

English language teaching has undergone massive changes in the past several decades in China (Jiang and Zhang, 2021; Yuan and Zhang, 2021; Chen et al., 2022; Liu and Chu, 2022; Sun and Zhang, 2022). University English teaching has been critiqued for its teacher-centered classrooms and dominance of the traditional Grammar-Translation teaching method (Liu and Xu, 2017; Wang and Zhang, 2021; Wu X. M. et al., 2021; Zhang and Zhang, 2021). This method requires students to have a good command of grammatical rules and sentence structures with a focus on vocabulary, translation, and written language (Richards and Rodgers, 2015; Richards, 2018; Sun and Zhang, 2021). Unfortunately, students instructed by this method are “deaf and dumb” English learners, which means that they gain insufficient listening and speaking skills and still have difficulty in verbal communication. In response to this issue in college English teaching, the Chinese Ministry of Education (hereafter MOE), has made massive top-down reforms for advancing English language teaching (ELT) (Zhang and Liu, 2014; Li et al., 2020; Teng and Zhang, 2020; Sun and Zhang, 2022). A recent reform is that the MOE has released the *College English Curriculum Requirements* (CECR) (Chinese Ministry of Education, 2007; see an updated version released in 2017), which is a mandate for college English teaching. Compared to the earlier *College English Syllabus*, the 2007 CECR accords its importance to developing students’ listening and speaking skills. It stipulates that college English teaching should be directed at cultivating students’ communicative competence and enhancing their ability to study independently, so as to meet the needs of China’s social development and international exchanges (Zhang and Liu, 2015). The CECR requires that College English teachers change themselves from solely using traditional teaching methods to embracing some new methods, including various manifestations of how communicative language teaching is done (e.g., task-based language teaching). Such a mandate is expected to enable teachers not only to teach linguistic knowledge but also to develop students’ communicative competence in using English (Zhang, 2021; Liu et al., 2022; Zhang and Liu, 2022). Ten years have passed and yet no large-scale studies so far have been conducted to investigate how Chinese University English teachers approach English teaching in their classrooms, especially how they deal with grammar instruction after the new curriculum reform was implemented. This study intends to fill this gap.

## Literature Review

### Teacher Cognition

Research into language teacher cognition emerged and flourished as the study of language teaching shifted its focus from a process-product approach to a teachers’ thinking paradigm (Clark and Peterson, 1986; Sun and Zhang, 2019, 2021, 2022; Gao et al., 2020; Gao and Zhang, 2020; Li, 2020). Borg (2003) thinks that “the unobservable cognitive dimension of teaching: what teachers know, believe, and think” (p. 81) is the knowledge base that is typically called teacher cognition. All teachers have their own thinking about teaching and learning, such cognition reflects their individual teaching philosophies (Pajares, 1992; Richards, 1996). Teacher cognition provides guidance to teachers’ decision-making by acting “as a filter through which a host of instructional judgements and decisions are made” (Fang, 1996, p. 51). Research into language education has also revealed that language teachers hold a complex set of beliefs about their students, teaching methods, and classroom decision-making (Barnard and Burns, 2012; Yuksel et al., 2021). Language teachers’ cognitions exert a strong influence on their pedagogical decision-making, instructional practices, and professional development (Borg, 2006, 2015), and is considered to be the guiding principles for them to implement their classroom teaching (Borg, 2006; Farrell and Bennis, 2013; Li, 2020; Zhang and Zhang, 2020; Wu D. A. et al., 2021). Given the diverse range of skills involved in language teaching, scholars have investigated teachers’ cognitions about discrete language skills (e.g., reading, writing, listening, speaking, grammar, and vocabulary). Due to the space constraint, we confine our discussion to grammar instruction in this article.

### Grammar Instruction

Grammar instruction in foreign/second language teaching has undergone great fluctuations over the past few decades in many parts of the world (Nassaji and Fotos, 2011; Nassaji, 2018; Cheng and Zhang, 2021; Nassaji and Kartchava, 2021; Zhang and Cheng, 2021). It was prioritized in typical traditional language teaching classrooms through the use of a grammar-translation method in the field of language teaching. Gradually, it was marginalized, especially when communicative approaches gained their prominence. In the last two decades researchers have proposed for a reconsideration of the significance of the role of grammar (Long, 1996; Schmidt, 2001), and the significance of grammar instruction was generally re-established (VanPatten, 1996; DeKeyser, 1998; Long, 2015). The focus has since shifted to how to teach grammar effectively (Long and Robinson, 1998; Borg, 1999; Ellis, 2001; Ellis and Natsuko, 2015). Unfortunately, given the complexity of language teaching itself, researchers have not reached any agreement on how teachers should approach grammar in the classroom (Ellis, 2006). Our search of the literature has enabled us to identify several recurring themes out of the available empirical studies. As can be anticipated, some of the themes overlap in content, despite the different labels used in these studies. These themes include: focus-on-formS instruction, focus-on-form instruction, deductive approaches to grammar instruction, inductive approaches to grammar instruction, using grammatical terminology, and grammatical drilling. In the following sections, we examine these themes in some detail.

#### Focus-on-FormS Instruction

Based on linguistic structuralism, focus-on-formS instruction emphasizes teaching language forms in isolation rather than the meanings they convey (Long, 1991; Ellis et al., 2002). The target language is divided into separate linguistic features, such as words, sentences, structures, grammar rules, functions, among other things. This type of instruction is mainly associated with synthetic classroom devices, including explicit grammar rules, repetition of model sentences, transformation exercises, and direct error correction. It primarily consists of work on the linguistic items with little opportunity for learners to use language for communication. Doughty and Williams (1998) point out that in focus-on-formS instruction learners engage in production activities ranging from mechanical to more communicative drills. These drills have the pitfall that too much attention to linguistic forms results in deliberate rather than automatic language use, which does not lead to natural communication. Despite its drawback, this kind of instruction is still used in teaching learners with simple English without much attention on communication. It is also adopted widely in many EFL contexts, especially in countries where examination-oriented English teaching is emphasized.

#### Focus-on-Form Instruction

The concept of focus-on-form instruction traces back to Long who firstly made a distinction between *focus-on-form* and *focus-on-formS* (Long, 1991). In Long’s view, focus-on-formS is a traditional approach, based on the assumption that language is made up of a series of grammatical forms that can be acquired successively, while focus-on-form draws learners’ attention to linguistic forms in the context of meaningful communication. The crucial distinction is that “focus on form entails a prerequisite engagement in meaning before attention to linguistic features can be expected to be effective” (Doughty and Williams, 1998, p. 3). The advantage of focus-on-form is that it solves the limitation that focus-on-formS instruction shows little concern with communicative use but still retains the strength of the traditional approach. Focus-on-form is associated with learner-centered approaches and happens when the learner attends to meaning and aims at resolving a communication problem (Nassaji and Fotos, 2011). It has been extensively applied in communicative language teaching classrooms (Lightbown and Spada, 1990; Zhang and Ben Said, 2014; Zhang and Rhimi, 2014; Long, 2015, 2016).

There were substantial studies on the effect of focus-on-formS and focus-on-form instruction and mixed results were obtained. Some studies demonstrated that focus-on-form instruction was more effective. For example, Burgess and Etherington (2002) reported on teachers’ attitudes to grammar and its teaching and learning within an EAP context. Results indicated that the majority of teachers appreciated the value of grammar (focus-on-formS) for their students and yet they also showed a favorable attitude to the focus-on-form approach. However, different research findings regarding the effectiveness of the two approaches were also noted. Norris and Ortega (2000) examined the effectiveness of grammar instruction by conducting a meta-analysis of experimental and quasi-experimental studies. They found that focus-on-formS and focus-on-form instructions were equally effective in facilitating language learning.

#### Deductive and Inductive Approaches

One of the controversial issues in grammar teaching centers on a dichotomy between deductive and inductive approaches. According to Thornbury (1999), a deductive approach begins with the teacher explicitly stating grammar rules or sentence patterns, which the learners then apply to practical use, while an inductive approach does not start with an explicit presentation of the rules; instead, learners are prompted in some way to discover the underlying patterns of the targeted structure and may possibly be required to formulate the rules that govern it.

It is contentious regarding which approach is more effective in facilitating the learning of a second/foreign language (Ellis, 2006, 2008). Empirical research investigating the relative effectiveness of deductive and inductive approaches of explicit grammar instruction has produced contrasting results. Some studies reported that the inductive approach led to higher gains in learning than did deductive instruction. For example, through a study of 26 university students learning French in an American university, Herron and Tomasello (1992) drew a conclusion that an inductive approach was more effective than a deductive approach. Other studies showed that deductive instruction was more effective. For instance, Erlam’s (2003) study revealed a significant advantage in her study of deductive instruction over the inductive instruction group when she investigated New Zealand secondary students learning Direct Object Pronouns in French as a second language by isolated grammar instruction, namely, the deductive approach vs. the inductive approach. However, no significant differences were found between these two types of instruction in terms of their relative effectiveness in the majority of studies. For instance, in order to explore the relative effectiveness of an inductive presentation and a deductive one either when difficult concepts are being learned or when the students are weak, Shaffer (1989) conducted a study on 319 students from three different high schools in the US. The results show no significant differences between the two forms of grammar presentation. In sum, the effectiveness of the deductive approach and the inductive approach in language teaching remains inconclusive.

#### Use of Grammatical Terminology

Whether integrating the use of grammatical terminology into grammar instruction second/foreign language education (L2) is also a point of controversy. Some researchers support the use of grammatical terminology in L2 learning. For instance, Hutchinson (1987) argued that in order to talk about a language easily and clearly, learners needed to be familiar with the metalanguage of grammar, i.e., using terminology. Metalanguage refers to “the names of parts of speech, tenses, etc.” (p. 14). He argued that terminology plays a role of “lubricant” in explaining grammatical knowledge to students. Therefore, using terminology is essential and should be integrated into classroom teaching and learning. In contrast, other researchers cast their doubts on the use of terminology in L2 teaching by presenting a range of reasons to avoid its use. Garrett (1986), for example, argued that knowing grammatical terminology by itself did not promote students’ language proficiency. He offered two reasons: (1) even the most familiar terms could be seriously misleading when they were used to explain grammar structures or rules; (2) grammatical terminology could not, of itself, evoke students’ understanding of the processing for the production of a grammatical structure.

#### Drilling

Drilling is a technique or tactics that has been used in L2 classrooms for many years, whose aim is for students to internalize grammatical structures or sentence patterns by repeating them until they are able to memorize them. Initially, drilling was a pedagogical method typically used in the audio-lingual method to language teaching with an emphasis on repeating structural patterns through oral practice in mechanical ways. Later, drilling evolved into any practice in language teaching classrooms which used specific language items in a controlled manner (Harmer, 2007). When teachers introduce new language items to their students, they tend to ask students to practice them either in some discrete sentences or in a real communicative context.

Drilling is widely used in teaching and learning new grammar points in L2 education. In order to instruct students to master these grammar points and apply them in their own language output, teachers drill their students in learning these grammar points. For example, Teik (2011) investigated pre-service teachers’ beliefs about the teaching and learning of grammar in Singapore after the implementation of the newly revised *English Language Syllabus* (Ministry of Education, 2010). More than half of the teachers who answered the questionnaire responded positively. They believed that drilling students in the patterns of grammar usage helped them remember the rules, and such findings echo what was reported in Goh et al., 2005).

### Teachers’ Cognitions About Grammar Instruction

Studies exploring teachers’ cognitions about grammar instruction in a range of L2 contexts have been reported recently. Sanchez and Borg (2014), for example, examined two experienced secondary school English teachers’ cognitions about grammar teaching in Argentina. Data were primarily gathered by some qualitative methods such as semi-structured interviews, classroom observations, and stimulated recall interviews. In the same vein, Nishimuro and Borg (2013) examined three experienced Japanese English teachers’ cognitions about, and practices in, teaching grammar. Their data were also elicited by qualitative methods including interviews, classroom observations, and stimulated recall interviews. Phipps and Borg (2009) explored the tensions between three experienced Turkish L2 teachers’ beliefs about grammar teaching and their practices by using interviews and classroom observations. These studies tended to use a triangulation of qualitative research methods to look into teachers’ cognitions about grammar teaching in multiple social contexts excluding China (cf. Gao and Zhang, 2020; Sun and Zhang, 2021).

In China, there have been some sporadic studies exploring EFL teachers’ cognitions. But most of them focused on teachers’ cognitions about L2 teaching in general. For example, three studies used qualitative methods for investigating the issue. Li (2013) investigated one experienced secondary school L2 English teacher’s beliefs and classroom practices as well as the relationship between them. Xiang and Borg (2014) examined Chinese College English teachers’ beliefs about effective language teaching. And Kang and Cheng (2014) made an in-depth case study on a novice middle school EFL teacher’s cognition development. As can be seen from our review above, research into teachers’ cognitions about grammar instruction on a large scale in the context of China is not documented.

## Method

In order to fill the research gap, this study aims to probe into a group of Chinese College L2 English teachers’ cognitions about grammar teaching on a large scale by developing and validating an instrument, the Teachers’ Cognitions about Grammar Teaching Questionnaire (TCAGTQ) in order to collection information on a larger scale (see Sun et al., 2016; Teng and Zhang, 2016). The key objective is to establish its reliability and validity for collecting information on teachers’ cognitions about grammar teaching in three distinct phases: (1) TCAGTQ development and initial validity; (2) confirmation and refinement of the modified TCAGTQ; (3) exploration of the correlations among different scale factors.

### Instrument Development and Initial Validity

In order to elicit Chinese L2 English teachers’ cognitions about grammar teaching, the TCAGTQ was developed with items adapted from Andrews (2003) (see Appendix). Andrews (2003) developed a belief inventory to investigate Hong Kong secondary school English teachers’ cognitions about grammar and grammar pedagogy. Teachers’ cognitions were categorized into six themes: Focus-on-formS instruction, focus-on-meaning instruction, the explicit and deductive approach, the inductive approach, the value of drilling, and the importance of using grammar terminology. The purpose of our study is to establish validity and reliability of the TCAGTQ with Chinese university L2 English teachers as respondents. It is worthy to note that some adaptations were made to Andrews’ (2003) questionnaire to cater to the educational context of mainland China and meet the purpose of the study. For example, users of Chinese usually avoid the absolute tone in their expressions and seldom use words like “all,” and “always.” Therefore, we deleted from the questionnaire items all such words that contain this kind of absolute tone. In the end a 35-item questionnaire was finally constructed. Similar to Andrews’ (2003) original questionnaire, six themes of teachers’ cognitions about grammar instruction were included in our reconstructed questionnaire: Focus-on-formS instruction (FoFs), focus-on-form instruction (FoF), the explicit and deductive approach (DA), the inductive approach (IA), the importance of grammatical terminology (GT), and the value of drilling (VD).

The TCAGTQ consisted of two sections. Section “Introduction” provided general information about participants’ background (e.g., age, gender, teaching experience, academic qualifications, and overseas studying experience). Section “Literature Review” contained 35 items designed to elicit responses to different statements about teaching grammar. A five-point Likert scale ranging from 1 (strong disagree) to 5 (strongly agree) was used for participants to indicate their agreement or disagreement. The Likert-type scale is a most widely used method of scale construction due to its simplicity, versatility, and practicality (Dörnyei, 2010). Participants were not required to judge the items but to indicate to what extent they agree or disagree with the items in the questionnaire according to their instincts. They could make a choice according to their own understanding. Since the targeted participants were university English teachers, the questionnaire was presented in English.

To ensure its content validity, the initial questionnaire was checked twice. First, four university English teachers were invited to examine the initial pool of questionnaire items, construct consistency, and the wording. They were then invited to check the suitability of the questionnaire in terms of its clarity and readability. After the revision of some questionnaire items, the questionnaire was finalized and regarded as being ready for use.

### The Participants

A total of 982 English teachers from universities or colleges in a northern province of China were invited to answer the TCAGTQ online through using Survey Monkeys. Altogether 529 teachers voluntarily completed the TCAGTQ, representing a response rate of 53.9%. Among them, 406 were female and 117 male. Apparently, female participants (76.7%) far outnumbered male ones (23.3%). In their ages, more than half of participants were aged from 31 to 35 (*n* = 265, 50.9%). A very small number of participants were aged from 20 to 25 (*n* = 10, 3.2%) and more than 45 (*n* = 38, 7.2%). Participants in other three age ranges were quite similar, and they were 26–30 (*n* = 63, 11.9%), 36–40 (*n* = 92, 17.4%) and 41–45 (*n* = 61, 11.5%). In terms of their current academic qualifications, the majority of participants had a Master’s degree (*n* = 458, 86.6%), and the others held a Bachelor’s degree (*n* = 36, 6.8%) and a Doctoral degree (*n* = 35, 6.6%). As for their teaching experience, most participants had 6–10 years’ teaching experience (*n* = 220, 41.6%) and more than 10 years’ teaching experience (*n* = 211, 39.9%), while approximately one fifth of the participants (*n* = 98, 18.5%) taught college English for less than 5 years. Finally, when participants were asked about whether they used to study English in English speaking countries, nearly half of participants gave a positive response (*n* = 266), representing 50.3%, and the duration of their overseas study were varied, whereas more than half of participants did not undergo overseas study yet (*n* = 263, 49.7%).

### Data Collection and Analysis

Before conducting factor analysis, we assessed whether the mean, variance, and coefficients of skewness and kurtosis were normally distributed. Descriptive statistics showed that the mean scores of all items ranged between 2.00 and 4.29 with the standard deviations from 0.755 to 1.322. The values for skewness and kurtosis of all items were between −1.360 and 0.524 and −1.247 and 3.569. According to Kline (2011), skewness coefficients with a range of no more than 3.0 and kurtosis coefficients with a range of no more than 8.0 are considered normally distributed data fits. The values in our study fall within the recommended range, suggesting univariate normality for factor analysis.

In order to develop and evaluate the TCAGTQ, Exploratory Factor Analyses (EFA) using SPSS were conducted on the first stratified random subsample (*n* = 314) to identify factors in TCAGTQ. Next, Confirmatory Factor Analyses (CFA) through structural equation modeling (SEM) were performed on the second stratified random subsample (*n* = 215) to check the reliability and construct validity. Finally, the score reliability of the final version of TCAGTQ was determined. Effect sizes were checked with reference to the recommendation in Wei et al. (2019).

## Results and Discussion

### Exploratory Factor Analyses

In order to identify the underlying structure of TCAGTQ, a principal component analysis based on a varimax rotation was performed on the first stratified random subsample (*n* = 314). The Kaiser-Meyer-Olkin (KMO) competency was measured to test the sample size validity statistically. Results show a KMO value of 0.850, suggesting that the data were suitable for structural detection. The Kaiser’s eigenvalues-greater-than-one (K1) rule and the scree plot were adopted as criteria to extract the number of factors. To interpret the factors, we opted for factor loadings larger than 0.40 (Field, 2009). Low factor loadings and cross-loadings (8 items) were excluded. As a result, a 6-factor solution with 27 items was accepted, which accounted for 50.55% of the total variance. The results are presented in Table 1. A variance value above 40% is considered sufficient for social science studies (Gorsuch, 1983). Therefore, the current total variance was within the acceptable limits. In addition, the six factors were found to be reliable as they were all above the threshold of Cronbach’s alpha 0.7 (DeVellis, 2012; see Table 1).

**TABLE 1:** Reliability of the TCAGTQ.

Factor	Item	Factor loadings						
		1	2	3	4	5	6	α
Focus on forms	FoFs 1	0.814						0.78
	FoFs 2	0.798						
	FoFs 3	0.726						
	FoFs 4	0.624						
	FoFs 5	0.555						
	FoFs 6	0.545						
Focus on form	FoF1		0.830					0.82
	FoF2		0.718					
	FoF3		0.716					
	FoF4		0.604					
	FoF5		0.584					
The deductive approach	DA1			0.794				0.74
	DA2			0.774				
	DA3			0.763				
	DA4			0.729				
	DA5			0.609				
The inductive approach	IA1				0.760			0.74
	IA2				0.723			
	IA3				0.693			
	IA4				0.675			
	IA5				0.670			
The use of metalanguage	GT1					0.868		0.72
	GT2					0.737		
	GT3					0.728		
Drilling	VD1						0.763	0.70
	VD2						0.739	
	VD3						0.601	

### Confirmatory Factor Analyses

**FIGURE 1:**
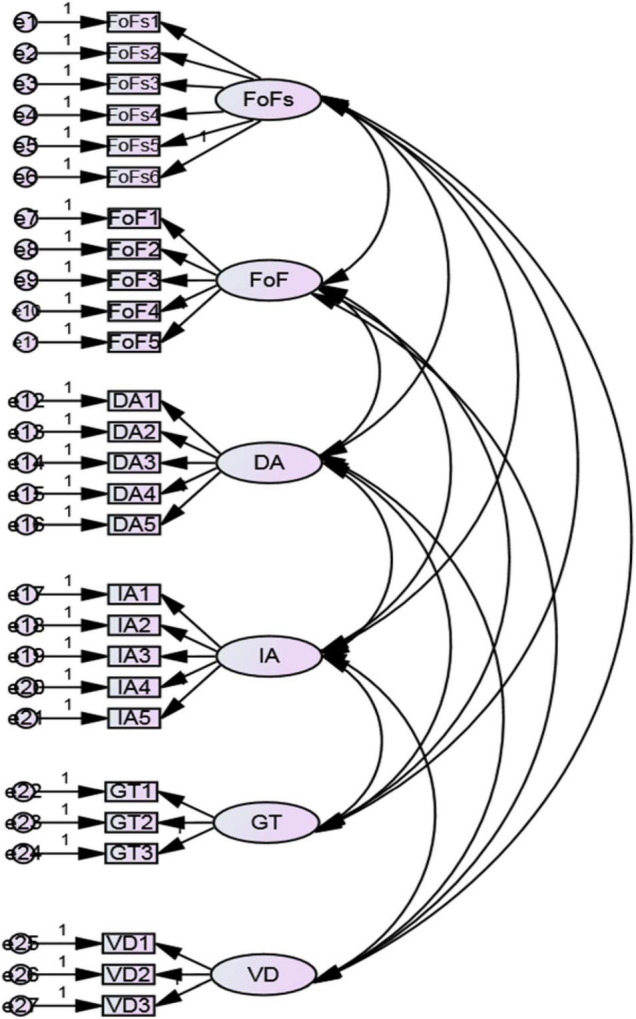
A six-factor model. *FoFs*, focus on forms instruction; *FoF*, focus on form instruction; *DA*, the deductive approach; *IA*, the inductive approach; *GT*, the importance of grammatical terminology; *VD*, the value of drilling.

In order to verify whether the factor constructs fitted our sample, CFA were performed with AMOS ver 0.23 based on the data from the second stratified random subsample (*n* = 215). According to Kline (2011), the Chi-square test (*x^2^ /df* ration), the goodness-of-fit index (GFI), the comparative fit index (CFI), the Tucker-Lewis index (TLI), the root mean square error of approximation (RMSEA), and the standardized root mean square residual (SRMR) are the essential model fit indices which should be included when reporting the results of CFA. The CFA results (x^2^/df = 2.025, CFI = .922, GFI = .901, TLI = .910, RMSEA = 0.556 and SRMR = .045) for the six-factor model with 27 items of our study revealed acceptable model-fit (see Figure 1), though TLI was below the threshold value of 0.95. Given the minimum item requirement for each factor and the sensitivity of TLI to misspecification and sample size (Fan and Sivo, 2007), no elimination of indicators was further conducted. This model was still accepted with good model fit (see Figure 1).

The reliability of each scale was determined by the internal consistency estimates of Cronbach’s alpha coefficient. The Cronbach’s alpha scores for all scales were satisfactory and greater than 0.7 (Field, 2009; see Table 1).

### Relationship Between Teachers’ Cognitions About Grammar Teaching Questionnaire Subscales

The predictive validity of TCAGTQ was tested through the correlations between the 6-factor construct. The nature of the relationships among subscales of TCAGTQ was revealed by the results of the bivariate correlations. Table 2 gives an overview of the correlations. The result shows that moderate correlations were noted among six subscales.

**TABLE 2:** Intercorrelations between TCAGTQ Subscales.

	FoFs	FoF	DA	IA	GT	VD
FoFs	1	0.056	0.569**	0.231**	0.462**	0.273**
FoF	0.056	1	0.264*	0.577**	0.044	0.115*
DA	0.569**	0.264*	1	0.349**	0.423**	0.328**
IA	0.231**	0.577**	0.349**	1	0.200**	0.168**
GT	0.462**	0.044	0.423**	0.200**	1	0.191**
VD	0.273**	0.115*	0.328**	0.168**	0.191**	1

**p ≤ 0.01 level (2-tailed) *p ≤ 0.05 level (2-tailed).

The purpose of the current study was to develop and validate a self-report questionnaire to assess EFL teachers’ perceptions about grammar teaching in Chinese universities. The factorial analyses through EFA and CFA with two stratified random samples produced sufficient evidence for the 6-factor construct, including focus-on-formS instruction, focus-on-form instruction, the deductive approach, the inductive approach, the use of grammatical terminology, and the value of drilling. The first theme, focus-on-formS instruction (FoFs) is similar to the traditional Grammar-Translation teaching method. Using this method, teachers give priority to grammar rules and linguistic features and emphasize accuracy more appropriate language use. The second theme, focus-on-form instruction (FoF) refers to a grammar teaching method whereby teachers not only highlight the accuracy in language use but also draw students’ attention to using language properly in communicative contexts. The third theme, using the deductive approach (DA), is a grammar teaching method in which teachers teach grammar rules to students directly and explicitly and ask students to practice them for practical language use. The fourth theme, using the inductive approach (IA), refers to a grammar teaching method where teachers do not present grammar rules to students, but instead they ask students to discover grammar rules or features. The fifth theme, giving importance to grammatical terminology (GT), explores teachers’ attitudes toward the use of grammatical terminology in grammar teaching. The sixth theme, according value to mechanic drills (VD), is teachers’ beliefs in learning grammar points through mechanic and formulaic drills.

The EFAs on the original TCAGTQ, which had 35 items, yielded a 6-factor solution with 27 items in the final version of the scale. That is to say, six themes of teachers’ cognitions about grammar teaching in the questionnaire were explored. Such findings echo Andrews’ (2003) questionnaire that six types of teachers’ beliefs about grammar teaching were examined in relation to Hong Kong secondary English teachers. The fact that six themes of teachers’ cognitions about grammar teaching were often examined is seen in many previous studies (Borg, 1999; Norris and Ortega, 2000; Burgess and Etherington, 2002; Erlam, 2003; Teik, 2011).

CFA was conducted to cross-validate six-factor structure generated from EFA. The results of CFAs supported the 6-factor TCAGTQ construct, including focus-on-formS instruction, focus-on-form instruction, the deductive approach, the inductive approach, the use of grammatical terminology, and the value of drilling. The correlations between the six themes of teachers’ cognitions about grammar teaching were also explored in order to examine the predictive validity of TCAGTQ. The result revealed the 6-factor model with good fit, confirming that the six themes of teachers’ cognitions were not only able to distinguish from one another but also inter-correlated on both conceptual and empirical bases.

## Conclusion and Implications

We developed and validated a self-report questionnaire that explored Chinese University EFL teachers’ cognitions about grammar teaching (TCAGTQ). Specifically, to elicit Chinese university EFL teachers’ cognitions about grammar teaching in terms of six themes, including focus-on-formS instruction, focus-on-form instruction, the deductive approach, the inductive approach, the use of grammatical terminology, and the value of drilling, a 6-factor model with good psychometric properties was generated through EFA and CFA. Results suggest good content validity and predictive validity of TCAGTQ.

Given the timeframe and the focus of this study, and despite limitations, an immediate implication of our study for English language teaching is that teachers can self-assess their cognitions about grammar teaching through this validated questionnaire, TCAGTQ. Based on their own discovery, teachers can then design their grammar lessons according to learner needs that would maximize student benefit. Such a self-reflective approach would also help teachers’ professional learning in the long run. Teacher might also find that using TCAGTQ with their students can be a mutually enriching experience for better provision of grammar instruction on the basis of negotiating a common ground upon which grammar teaching can be effectively conducted.

It needs to be pointed out that like many studies, our study also suffers limitations. First, the participants in this study were only recruited from one province of China. If teachers from other areas, especially those from municipal cities were involved in the study, a more holistic understanding of teachers might be better obtained (Sun et al., 2016). Second, the sample size is not sufficiently large, which restricts the generalizability of the results (Dörnyei, 2010). Therefore, continuous efforts in these aspects need to be made in future research.

## Data Availability Statement

The original contributions presented in this study are included in the article/supplementary material, further inquiries can be directed to the corresponding author.

## Ethics Statement

The studies involving human participants were reviewed and approved by the University of Auckland Human Ethics Committee. The patients/participants provided their written informed consent to participate in this study.

## Author Contributions

Both authors listed have made a substantial, direct, and intellectual contribution to the work, and approved it for publication.

## Conflict of Interest

The authors declare that the research was conducted in the absence of any commercial or financial relationships that could be construed as a potential conflict of interest.

## Publisher’s Note

All claims expressed in this article are solely those of the authors and do not necessarily represent those of their affiliated organizations, or those of the publisher, the editors and the reviewers. Any product that may be evaluated in this article, or claim that may be made by its manufacturer, is not guaranteed or endorsed by the publisher.

## Appendix

### Factors and Items of the Teachers’ Cognitions About Grammar Teaching Questionnaire (TCAGTQ)

*Focus-on-formS instruction (six items)*

1. If students memorize rules and facts about grammar, it will help them to produce correct language in spontaneous situations.
2. Students should be encouraged to speak/write accurately from the beginning.
3. Students need to be consciously aware of a structure’s form and its function before they can use it proficiently.
4. When use language to communicate in English, it is more important to be grammatically accurate than socially appropriate.
5. Accuracy, or correctness in linguistic form, is a primary aim in grammar teaching.
6. Teachers should focus more on the structure and form than meaning.

*Focus-on-form instruction (five items)*

1. Grammar teaching should focus on the meaning of structures and their use in context.
2. Students learn grammar best through exposure to language in natural context.
3. Focusing students’ attention on forms is a necessary but not sufficient condition for the acquisition of grammar.
4. Thinking about the grammar rules while talking prevents students from communicating fluently.
5. New grammatical points should be presented and practiced in situations.

*The deductive approach (five items)*

1. Grammar should be taught in an explicit or direct way.
2. Teachers should teach simple grammatical structures before more complex ones.
3. Teaching grammar in different units leads to language knowledge which students can use in natural contexts later.
4. Teachers should begin teaching a new grammar point by explaining the rule.
5. It is best to give the grammatical explanation first and then practice the rule.

*The inductive approach (five items)*

1. Having students figure out grammatical rules can help them increase their awareness of English.
2. Students should be encouraged to create language by a process of trial and error.
3. Teachers should begin teaching a new grammar point by giving examples.
4. Grammar explanations should be avoided by the teacher.
5. Teachers should help students to work out grammar rules for themselves.

*The importance of grammatical terminology (three items)*

1. Teachers should use grammatical terms to explain grammar rules to students.
2. Students should be able to use the common grammatical terms in English correctly when discussing grammar.
3. Students should understand the common grammatical terms in English.

*The value of drilling (three items)*

1. Drilling and memorization are essential to the successful learning of new language forms.
2. Mechanical drilling is of no value in English teaching.
3. Teachers should ask students to practice new grammatical structures.
